# Complete chloroplast genome of Ulleung Island endemic, *Epilobium ulleungensis* (Onagraceae), in Korea

**DOI:** 10.1080/23802359.2018.1481796

**Published:** 2018-06-27

**Authors:** Ji Young Yang, Jae-Min Chung, Seung-Chul Kim

**Affiliations:** a Research Institute for Dok-do and Ulleung-do Island, Kyungpook National University, Daegu, Republic of Korea;; b Korea National Arboretum, Pocheon, Republic of Korea;; c Department of Biological Sciences, Sungkyunkwan University, Suwon, Republic of Korea

**Keywords:** Chloroplast genome, Korean endemic, *Epilobium ulleungensis*, Ulleung Island, Onagraceae

## Abstract

The first complete chloroplast genome sequences of Korean endemic *Epilobium* in Ulleung Island, *Epilobium ulleungensis*, were reported in this study. The *E. ulleungensis* plastome was 160,912 bp long, with the large single copy (LSC) region of 88,915 bp, the small single copy (SSC) region of 17,327 bp, and two inverted repeat (IR) regions of 27,335 bp. The plastome contained 131 genes, including 84 protein-coding, eight ribosomal RNA, and 37 transfer RNA genes. The overall GC content was 36.5%. Phylogenetic analysis of nine representative plastomes within the family Onagraceae suggests strongly that *E. ulleungensis* is sister to the clade containing species of *Oenothera* in tribe Onagreae.

Owing to its remarkable diversity in morphology, ecology, and cytology, the genus *Epilobium* L., with approximately 175 species, represents the largest group in Onagraceae and is mainly distributed in temperate regions (Raven 1988; Hoch and Raven 1992; Baum et al. 1994). As for infrageneric classification system, eight highly distinctive sections have been proposed and recent molecular phylogenetic study recognized two main clades, i.e. sect. *Epilobium* and the ‘xerophytic’ clade (six sections included), and sect. *Chamaenerion* being the earliest diverged lineage within the genus (Raven 197,6, 1988; Baum et al. 1994). Of eight sections delimited based on morphological and cytological characters (Raven 1976), sect. *Eplilobium* is the largest one (ca. 150 spp.) with major radiation in Australasia and smaller ones in temperate South America and southern Africa (Raven and Raven 1976; Baum et al. 1994). Although preliminary nrDNA ITS phylogeny within the genus was performed and infrageneric classification system was evaluated (Baum et al. 1994), several questions (e.g. relationship within the ‘xerophytic’ clade and sectional relationship among *Boisduvalia*, *Zauschneria*, and *Currania*) require additional study based on extensive sampling within the genus and additional plastid and independent nuclear markers. In Korea, a total of 12 species of *Epilobium* are currently recognized (National List of Species of Korea 2018), including one recently described insular endemic *E. ulleungensis* on Ulleung Island (Chung et al. 2017). Neither phylogenetic relationships nor complete chloroplast genome sequences is available among species of *Epilobium* in Korea. Therefore, in this study, we sequenced the plastome of *E*. *ulleungensis* and compared it to other plastomes in the family Onagraceae.

Total DNA (Voucher specimen: JMC15101, KH) was isolated using the DNeasy plant Mini Kit (Quiagen, Carlsbad, CA) and sequenced by the Illumina HiSeq 4000 (Illumina Inc., San Diego, CA). A total of 11,439,577 paired-end reads were obtained and assembled *de novo* with Velvet v. 1.2.10 using multiple *k*-mers (Zerbino and Birney 2008). The tRNAs were confirmed using tRNAsacn-SE (Lowe and Eddy 1997). The total plastome length of *E*. *ulleungensis* (MH198310) was 160,912 bp, with large single copy (LSC; 88,915 bp), small single copy (SSC; 17,327 bp), and two inverted repeats (IRa and IRb; 27,335 bp each). The overall GC content was 38.2% (LSC, 36.3%; SSC, 33.2%; IRs, 42.8%) and the plastome contained 131 genes, including 84 protein-coding, eight rRNA, and 37 tRNA genes. A total of 18 genes were duplicated in the inverted repeat regions, including seven tRNA, four rRNA, and seven protein coding genes including *ndh*F gene. The complete *ycf*1 gene was located in SSC and the *inf*A gene located in LSC became a pseudogene.

To confirm the phylogenetic position of *E. ulleungensis*, nine representative plastomes of Onagraceae and two outgroup species from Lythraceae were aligned using MAFFT v.7 (Katoh and Standley 2013) and maximum likelihood (ML) analysis was conducted based on the concatenated 77 coding genes using IQ-TREE v.1.4.2 (Nguyen et al. 2015). The ML tree (Figure 1) strongly suggested that *E. ulleungensis* (tribe Epilobieae) is sister to the clade containing species of *Oenothera* (tribe Onagreae).

**Figure 1.**
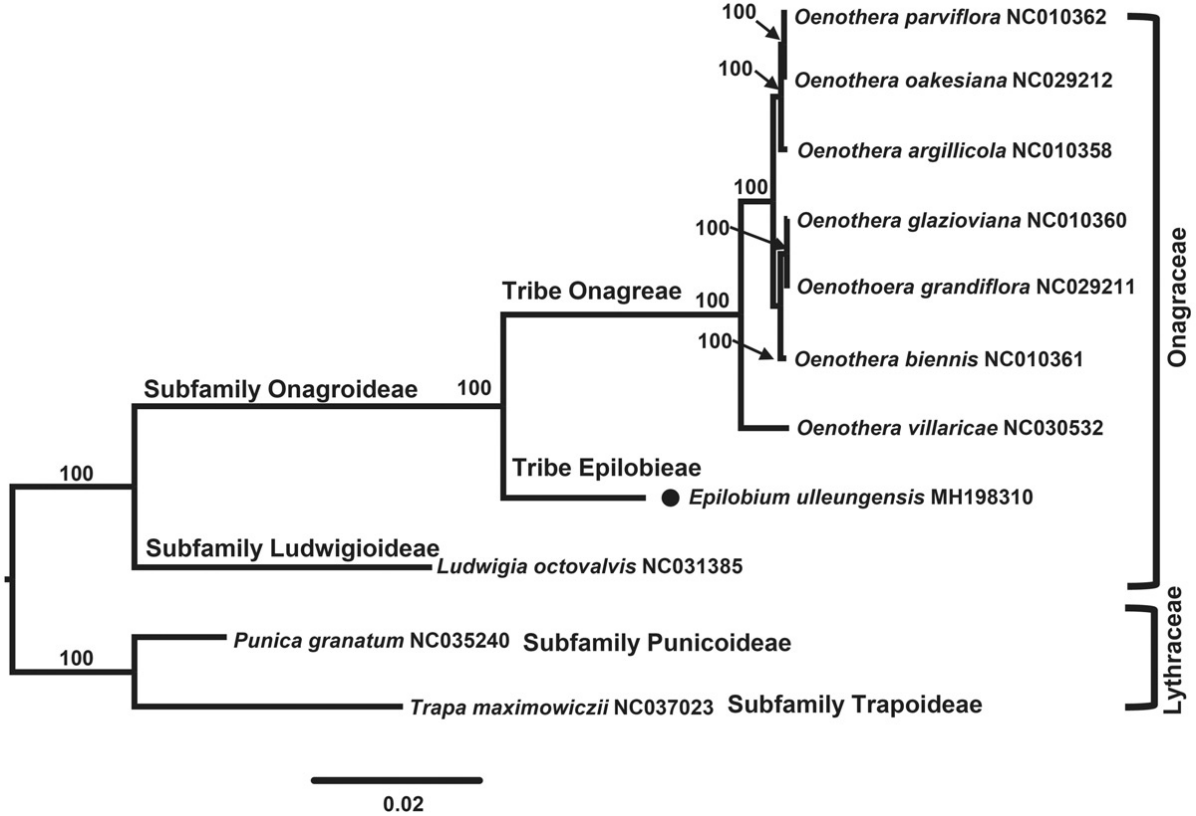
Maximum-likelihood (ML) tree based on 77 protein-coding genes in the nine representative plastomes of Onagraceae. The bootstrap value based on 1000 replicates is shown on each node.
